# Equitable and Holistic Public Health Measures During the Singaporean COVID-19 Pandemic

**DOI:** 10.5334/aogh.3244

**Published:** 2021-05-19

**Authors:** Samuel S. Y. Wang, Winnie Z. Y. Teo

**Affiliations:** 1 Fast Program, Alexandra Hospital, National University Hospital System, Singapore; 2 Department of Haematology-Oncology, National University Cancer Institute Singapore (NCIS), National University Health System, Singapore

## Abstract

This Covid-19 pandemic has been a trying time for all countries, governments, societies, and individuals. The physical, social, and organizational infrastructure of healthcare systems across the world is being stressed. This pandemic has highlighted that the healthcare of the country is as strong as its weakest link and that no aspect of life, be it social or economic, is spared from this pandemic.

The authors would like to highlight some of the lessons learned from Singapores management of the Covid-19 pandemic. During the Singaporean Covid-19 pandemic, public health policy planning was all encompassing in its coverage, involving various stakeholders in government and society. The important role of individuals, governments, industry, and primary healthcare practitioners when tackling COVID-19 are highlighted. Singapores management of the Covid-19 pandemic involved an approach that involved the whole of society, with a particular focus on supporting the vulnerable foreign worker population, which formed the majority of Covid-19 cases in the country. Hopefully amidst the trying times, valuable lessons are learnt that will be etched into medical history and collective memory. We hope to highlight these lessons for future generations, both for members of the public and fellow healthcare practitioners.

The Covid-19 pandemic has stressed healthcare infrastructure globally. Hopefully, through this experience healthcare systems will gather lessons to enable future generations to face pandemics. A particularly important lesson is that during a pandemic, a countrys healthcare is as strong as its weakest link. Moreover, no aspects of life, be them social or economic, are spared from this pandemic. Therefore, public health policy planning for pandemic preparation must be equitable in access and all-encompassing in coverage [1]. No member of society should be left out, and everyone has a specific role to play [1]. Tackling the pandemic involves breaking the chain of infection through social distancing, testing, and quarantine in an attempt to not overwhelm the health services and developing effective vaccines.

Mask wearing is an integral part of controlling Covid-19 infections through a combination of source control and personal protection for the mask wearer. Masks are mandatory in all public settings in Singapore. Despite the lack of effective vaccines available in Singapore at the height of the pandemic, the efforts of testing, quarantine, and social distancing have managed to stabilize infection rates and protect the healthcare systems from being overwhelmed. A particular lesson to draw from Singapores experience with Covid-19 would be to ensure that healthcare access remains equitable; it has been noted that vulnerable populations bear a disproportionate burden of Covid-19 infections and also are at risk of further propagating the pandemic.

## Unique Challenge of the Singaporean Covid-19 Pandemic

Covid-19 cases in Singapore have decreased from their high in April 2020. The Singaporean Covid-19 pandemic is interesting due to how the cases have been classified, namely foreign dormitory worker (FW) cases and community cases. Back in May 2020, community cases were under control, with a minimal number of imported cases and a daily average of single-digit cases, while the bulk of the daily infections were FW cases. The factors responsible for such a severe outbreak amongst FWs can be attributed to foreign dormitory workers being a marginalised and economically vulnerable population living in overcrowded and less sanitary accommodations. This compromises social distancing effectiveness and facilitates SARS-CoV-2 infections. A key nexus of infections was construction sites, where safe distancing practices were difficult to enforce, and the mixing of FW from various dormitories helped to drive the outbreak. Poor health literacy and education levels, plus cultural and language barriers, impeded effective communication of public healthcare policies. FWs also underreported symptoms or avoided medical help, fearing job/income loss. An interesting point about the FW outbreak is that it mirrors challenges observed in outbreaks throughout the developing world, but in the context of a highly urbanised and developed country. These basic challenges include language/cultural barriers, healthcare literacy, and overcrowded/unsanitary living conditions.

## Plight of Foreign Worker Populations in Singapore

It is well known that marginalised and economically vulnerable populations can potentially become infection clusters during a pandemic [2]. FWs are considered a vulnerable population due to existing poor health literacy, education level, and cultural and language barriers; this impacts their healthcare access. Prior to the pandemic, FWs were also living in unsanitary and overcrowded accommodations compared to the majority of the Singaporean population. Due to the poor hygiene and cramped living conditions, SARS-CoV-2 infections were common amongst FWs [3]. In Singapore they formed the majority of the infected and hospitalized population. Unfortunately, as part of infection control measures, FWs were segregated within their dormitories away from the majority of the population, which had the unintended effect of reinforcing negative stereotypes of FWs and promoting further social marginalization. Moreover, due to their FW status and despite bearing the brunt of the pandemic, the level of medical, financial, and social support provided to them was not equivalent to that of Singaporean citizens.

With the ongoing pandemic requiring social distancing measures, work resumption has been hampered. However due to the type of work FWs perform, they are often provided minimal worker compensation/unemployment benefits should they be unemployed from the tighter social distancing policies. Therefore, workers are often caught between sacrificing their income/economic livelihood and public health policy. Hence, many may try to continue working despite public health policies, which compromises social distancing effectiveness. They may also underreport symptoms or not seek medical help, fearing work cessation and job/income loss.

Other issues include poor health literacy, education level, and cultural and language barriers. This may lead to difficulty in communicating public healthcare policies. Additionally, communication barriers due to language may impede direct clinical care. The communication barriers may also generate fear of discrimination, which may alter health-seeking behaviour by these populations. Another potential problem is that such workers are foreigners and are potentially undocumented migrants, leading them being overlooked by healthcare planners [3].

Therefore, to address these challenges, multiple public health and economic measures must be undertaken to support this often-marginalized population. Firstly, establish a suitable housing environment to improve personal hygiene and overcrowding. Next, provide financial/economic benefits to encourage social distancing and lockdown compliance. Raising healthcare literacy through culturally and language-appropriate communication of healthcare policies is especially needed for this population. Singaporean officials tried their best in attempting to address the above issues despite the ongoing pandemic.

## Ensuring Infection Control and Equitable Healthcare Access

At a broad community level, public hygiene was strongly reinforced through the public media. This approach included regular handwashing, cough hygiene, donning of face masks, and even taking appropriate sick leave when unwell [4]. Mask wearing was made mandatory in all public settings and enforced by government officers. Masks were also made readily available through both government and non-government organizations to all Singapore residents as part of a public health measure. Additionally, at the peak of the pandemic in Singapore, non-essential economic activity was halted while key economic sectors were digitalized. This was done to enable the general population to effectively practice social distancing.

These measures helped to break the chain of transmission and flatten the curve slowing the pandemics progress, thereby buying precious time for Singaporean healthcare institutions to respond to the pandemic by upscaling SARS-CoV-2 testing capacity. This strategy enabled quick identification of asymptomatic/symptomatic infected individuals through aggressive testing of individuals with SARS-CoV-2 exposure. These data helped healthcare planners to identify infection patterns/clusters and proactively enact measures to stop them.

When tackling the FW outbreak, FWs in essential services were first separated and relocated to new self-isolation facilities away from infected dormitories. This measure aimed to prevent further infections and also preserve the FW manpower pool in essential services, which are key to running the country. Singapore has 261,900 FWs staying in 42 FW dormitories. To house the large FW population, with rudimentary but relatively effective monitoring and healthcare, community care facilities were rapidly developed using existing exhibition centres. This more than doubled Singapores hospital bed capacity. Next, an aggressive campaign of testing symptomatic/asymptomatic workers in the dormitories was undertaken to identify infected FWs. The infected FWs who were older, had comorbidities, and were symptomatic for severe Covid-19with dyspnoea and worsening feverswere admitted to acute hospitals for observation during the Covid-19 danger window. Once observed to be clinically improving, they were transferred to community care facilities for further observation until deemed to be non-infective either by a future double negative Covid-19 PCR nasopharyngeal swab or by duration of illness. In summary, greater equitable access to healthcare, clean sanitation, masks, and sick leave were crucial in breaking the chain of infection and establishing infection control [4].

## Maintaining Control Over the Covid-19 Pandemic

To maintain the low levels of Covid-19 infection within the community, public policy decisions were primarily made from a public health perspective guided by medical evidence. These clear, consistent, and evidence-based messages reduced healthcare literacy barriers and enabled the public to trust and comply with public health policies [5]. Frequent updates regarding the pandemics evolving nature also helped to dispel misinformation.

Increasing healthcare literacy and combatting misinformation were also important goals. Achieving these goals would ensure health-conscious behaviours are sustained and assist with future infection control and minimization of healthcare system burden [6]. Healthcare literacy was promoted throughout the community by the use of social and traditional media platforms with consistent and evidence-based information. There was greater understanding and compliance to limit the number of visitors at hospitals and nursing homes for infection control [7]. The population was also advised to visit with primary healthcare practitioners instead of going to acute hospitals in order to conserve healthcare resources and minimize infection risk. Individuals could also establish advanced medical directives regarding the extent of care for themselves/loved ones if they were advanced in age or had a life-limiting/debilitating illness. This approach intended to minimize rushing tough decisions regarding invasive medical treatments, in order to reduce psychological trauma for patients, family, and medical staff. With the extent of care established, resource planning and allocation could also be better streamlined.

A particular emphasis was made to reach out to FWs through community leaders or healthcare staff who were able to communicate with them. FWs often have poor healthcare literacy and, in conjunction with the sensationalized Covid-19 misinformation on social media, this might generate anxiety and fear amongst FWs, which might complicate public health efforts [8]. With the assistance of medical staff who could speak the FWs language, community leaders generated Covid-19 informational resources for the FWs by ensuring clear lines of communication between public health officials and FWs.5 Even with a reduction in the number of FW Covid-19 cases, continued social distancing and isolation amongst FWto break the chain of infectionis still important to prevent the FW cases from cross infecting and spilling over into the wider community. To assist the FWs, their salaries were subsidized and their daily living needs supported by the Singaporean government.

Despite controlling the pandemic, the government has been reluctant to fully normalize social distancing and mask-wearing rules, due to concerns of potential spikes of infections in urban contexts. To further enhance contact-tracing efforts, the government developed and encouraged the uptake of tracing applications/software such as the TraceTogether and SafeEntry national digital check-in system. These measures would facilitate contact tracing of exposed individuals should a positive Covid-19 case be identified.

Primary healthcare also played a key role in controlling the pandemic, because general practitioner (GP) clinics form the cornerstone of early identification and isolation of suspect cases within a vast patient pool. GP clinics were often the first point of contact for most undifferentiated cases, due to the high volume of patient flow and the non-specific nature of early Covid-19 infection [9]. To ensure the safety of staff and non-Covid-19 patients, GP clinics have dedicated isolation rooms, easy availability of masks to patients and personal protective equipment for staff. These measures aim at avoiding the development of clusters of infections within GP clinics [9]. These clinics can engage in follow-up care via telemedicine consults with their patients to reduce healthcare burden and risk of Covid-19 transmission. Additionally, with their long-established patient relationship, GPs can serve as healthcare educators in disadvantaged communities, dispelling Covid-19 myths while providing psychosocial counselling to ameliorate the pandemics mental health effects. Seasonal vaccinations through GPs should be increased to minimize hospital admissions and doctor visits, which are already stretched during the pandemic [10].

## Leadership Role of Government and Corporations During the Pandemic

Within the government, a dedicated Covid-19 multi-ministry taskforce was established to coordinate public policy to address the pandemics medical, social, and economic impact [11]. For example, the ministry of defence provided manpower for contact tracing and enforcement of public health policies. The ministry of finance ensured financial support to companies/individuals such that their livelihood was not compromised by public health measures [11]. Disadvantaged communities/individuals were prioritised when receiving financial aid because this pandemic has disproportionately affected their livelihood [11]. Prior to the pandemic, their low income left them with limited financial reserves in the event of unemployment [11]. Moreover, social distancing/lockdown policies disproportionately affect industries that support disadvantaged communities/individuals.

The multi-ministry taskforce acted as an independent authority overseeing deployment of the nations medical resources, such as medical personnel, personal protective equipment, and ventilators, to the areas of greatest need, ensuring equitable access [11,12]. This strategy reduced conflict amongst healthcare institutions for access to medical resources. An initiative was run to recruit retired healthcare personnel, streamline existing healthcare training, and also retrain non-healthcare professionals in the community to assist by providing basic care services.

Much of Singapores government policies were coordinated with corporations and industry [11]. The Singaporean media industry assisted in promoting health literacy through timely updates of latest health advisories from the World Health Organization and the Ministry of Health while reminding viewers to frequently cross check unverified Covid-19 claims [13]. Many local pharmaceutical, biotechnological, and medical technological companies collaborated with the government and research institutions to boost basic science and clinical trial research for the development of new vaccines and Covid-19 therapeutics [12]. The government provided the research funding while corporations and higher learning institutes offered the lab expertise and resources, and allowed for the compassionate use of drugs under development.

## Collective Memory from the Singaporean Experience

Finally, once the pandemic has passed, everyone has a collective responsibility to form a new normal in order to prevent future pandemics. Post-pandemic, individuals should maintain their health-conscious behaviours, healthcare literacy, and individual hygiene. Governments should develop contingency plans, stockpile key medical resources, and invest in public health infrastructure, such as disease surveillance, contact tracing, and testing. Other initiatives can include increasing vaccination rates, elevating public hygiene standards, and improving public health literacy. The biotech/pharmaceutical/medical technology industries should collaborate with governments to invest more resources into developing tests and treatments for emerging infections. At the same time, the media and tech industry should actively police content uploaded onto their networks to limit transmission of false information. Finally, GPs have an important role in serving at the front line of the Covid-19 pandemic; they are critical to early identification and isolation of suspect cases, ensuring continuity of care for non-Covid-19 patients, and serving as patients healthcare educator/mental health counsellors.

These policies and initiatives are summarised in ***Table 1***. They will collectively provide immunological memory, by ensuring that the wisdom of previous generations gained from a pandemic is preserved [14]. This pandemic has served as a painful inoculation to people and governments, while stimulating a social immunization that hopefully will minimize the risk of future pandemics.

**Table 1:** Summary of various roles that different members of society have to play when combating the Covid-19 pandemic.


PUBLIC HEALTH STAKEHOLDERS			

INDIVIDUAL	GOVERNMENT	INDUSTRY	PRIMARY HEALTHCARE PRACTITIONER

Maintaining individual health and hygiene Regular hand hygiene Cough hygiene Donning of face masks Taking appropriate sick leave Seasonal flu vaccination	Leadership Providing a voice of reason through consulting and listening to medical/healthcare professionals Regular public updates regarding the pandemics evolving nature Dispelling myths and health misinformation Building up public confidence in the countrys pandemic response Coordinate a society-wide approach when combating the pandemic through multi-ministry public policies Independent and centralised authority coordinating the deployment of the nations medical resource to areas of need Liaising with the private sector to coordinate private resources to assist in combating the pandemic Retrain other parts of the labour force to assist the healthcare industry Coordinate responses and act in solidarity with healthcare organizations, unions (i.e., World Health Organization, European Union, Association for South East Asian Nations)	Media industry Increase availability and access to accurate health information Police sources of health misinformation	Cornerstone of early identification and isolation of suspect cases Engage in telemedicine consults to reduce healthcare burden and risk of Covid-19 transmission Healthcare educators and mental health counsellors
Healthcare literacy Cross check sources of information Avoid spreading health misinformation Verify health claims against trusted health and scientific authorities		Pharmaceutical/biotechnology/medical technology industry Collaborate with government and private research institutes to perform basic science and clinical trial research Increase availability of compassionate use drugs, vaccines and diagnostic kits Increase production of medications, vaccines, and diagnostic kits	
Health-conscious behaviours Limit the number of visitors to healthcare facilities Visit the primary healthcare practitioner when not acutely unwell Consider advanced medical directives in frail individuals			

